# Joint Categories of Perceived Stress and Subjective Body-Shape Perception and Health-Related Outcomes Among Adolescents

**DOI:** 10.3390/children13101391

**Published:** 2026-10-13

**Authors:** Peng Zhou, Jiyoung Lee, Yonghwan Kim, Yoonjung Park

**Affiliations:** 1 School of Physical Education, Kashi University, Kashi City 844000, China; 20251019@ksu.edu.cn; 2 Department of Physical Education, Kangwon National University, Gangneung Campus, Gangneung 25457, Republic of Korea; kimyh@kangwon.ac.kr; 3 Laboratory of Integrative Physiology, Department of Health and Human Performance, University of Houston, Houston, TX 77204, USA

**Keywords:** adolescents, perceived stress, subjective body-shape perception, body mass index, self-rated health, mental health

## Abstract

**Highlights:**

**What are the main findings?**

- Adolescents with high perceived stress and self-perceived overweight showed higher odds of depressive symptoms, poor self-rated health, and alcohol experience than those with low stress and self-perceived non-overweight.
- Adolescents with objective non-overweight but self-perceived overweight had higher odds of high perceived stress than those with concordant non-overweight status.

**What are the implications of the main findings?**

- Joint consideration of perceived stress and subjective body-shape perception may provide a more comprehensive description of health-related characteristics among adolescents.
- Assessing discrepancies between objective weight status and subjective body-shape perception may offer additional insights into perceived stress during adolescence.

**Abstract:**

**Background**: Perceived stress and subjective body-shape perception may jointly characterize health-related conditions during adolescence. This study examined health-related outcomes across joint categories of perceived stress and subjective body-shape perception and compared perceived stress across combinations of objective and subjective weight status. **Methods**: This cross-sectional study included 1387 adolescents aged 12–18 years from the 2021–2024 Korea National Health and Nutrition Examination Survey. Survey-weighted multivariable logistic regression was used to examine depressive symptoms, poor self-rated health, and alcohol experience across four joint categories defined by perceived stress and self-perceived body shape, with adjustment for age, sex, household income, and body mass index. Objective weight status and self-perceived body shape were also combined into four categories to compare the odds of high perceived stress. Random Forest and SHapley Additive exPlanations (SHAP) analyses were additionally performed as exploratory cross-sectional classification analyses. **Results**: Compared with the low-stress, self-perceived non-overweight category, the high-stress, self-perceived overweight category showed higher odds of depressive symptoms (AOR = 15.96; 95% CI, 6.52–39.10), poor self-rated health (AOR = 7.55; 95% CI, 3.22–17.73), and alcohol experience (AOR = 3.04; 95% CI, 1.31–7.07). In the objective–subjective weight-status analysis, adolescents with objective non-overweight and self-perceived overweight showed higher odds of high perceived stress than those with concordant non-overweight status (AOR = 1.90; 95% CI, 1.27–2.86). The exploratory Random Forest model showed modest cross-sectional classification performance (ROC-AUC = 0.615; PR-AUC = 0.438), with depressive symptoms showing the largest SHAP contribution among the displayed features. **Conclusions**: Health-related outcomes differed across joint categories of perceived stress and self-perceived body-shape perception. Adolescents with objective non-overweight but self-perceived overweight also showed higher odds of high perceived stress than those with concordant non-overweight status. These findings highlight the relevance of considering perceived stress, subjective body-shape perception, and objective weight status together when describing adolescent health-related characteristics.

## 1. Introduction

Adolescence is a critical developmental period characterized by substantial physical, psychological, and social changes that may influence health and well-being [1,2]. Persistent or high levels of stress during this period may lead not only to mental health problems such as depression but also to poor self-rated health and health-risk behaviors such as alcohol use and smoking [3].

Among these factors, subjective body shape should be considered separately from objective weight status when understanding stress and psychological health in adolescents. During puberty, rapid changes in body weight and composition occur alongside increased sensitivity to peer and socioculturally shaped appearance standards, which may result in discordance between perceived body shape and actual weight status [4]. In a study of 72,825 U.S. adolescents, 35.4% of girls perceived themselves as overweight, whereas 24.2% were actually overweight; among boys, the corresponding proportions were 23.7% and 32.1%, respectively. These patterns of discordance also differed by sex and race [5].

The discrepancy between objective weight status and subjective body shape provides another perspective for understanding adolescent mental health. In a previous study, 55.0% of girls who were not objectively overweight but perceived themselves as overweight reported high levels of mental distress [6]. In addition, perceiving oneself as overweight during adolescence was associated with higher depressive and anxiety symptoms in early adulthood [7], and another adolescent study found that subjective weight perception explained internalizing and externalizing problem behaviors better than objective BMI [8].

Overestimation and underestimation reflect distinct forms of discordance between objective weight status and subjective body-shape perception. Such discordance is common during adolescence and may differ by sex, with girls more frequently overestimating and boys more frequently underestimating their weight status [9,10]. Previous studies among Korean adolescents have shown that weight misperception is related to depressive symptoms and other mental health characteristics, although the patterns may differ according to sex and the direction of misperception [4]. Notably, weight overestimation has been associated with perceived stress and other adverse mental health characteristics among Korean adolescents [11,12]. These findings support distinguishing overestimation from underestimation and considering both objective weight status and subjective body-shape perception when examining adolescent mental health.

However, previous studies have mainly examined individual associations among BMI, obesity, weight perception, and depressive symptoms. Differences in depressive symptoms according to weight status may be partly explained by self-perceived overweight perception, weight concerns, and dietary restraint [13]. In contrast, relatively few studies have combined adolescent stress level and body-shape perception into a single joint category and evaluated depressive symptoms, self-rated health, and health-risk behaviors together. Adolescent stress may also coexist with various lifestyle factors, including physical activity, alcohol use and smoking, meal frequency, and nutrient intake, with potentially complex and nonlinear relationships among these factors. Such characteristics may limit the ability of conventional group comparisons and regression analyses to determine the relative contribution of each factor. Therefore, SHapley Additive exPlanations (SHAP) was added as an exploratory complement to the regression analyses to characterize the relative contributions of multiple health, lifestyle, anthropometric, and nutritional features within a single classification framework, rather than to estimate independent associations or causal effects.

Accordingly, the primary objective of this study was to examine differences in prespecified health-related outcomes, including depressive symptoms, poor self-rated health, and alcohol experience, across four joint categories defined by perceived stress and self-perceived body shape among adolescents. The secondary objective was to compare the odds of high perceived stress across four observed combinations of objective weight status and self-perceived body shape. This joint-category approach was used to characterize adolescents with different combinations of stress and body-shape perception within a common framework, rather than to test statistical interaction or synergistic effects. As an exploratory objective, Random Forest and SHAP analyses were used to examine feature contributions within a cross-sectional classification model of high perceived stress.

## 2. Materials and Methods

### 2.1. Study Design and Participants

**Figure 1:**
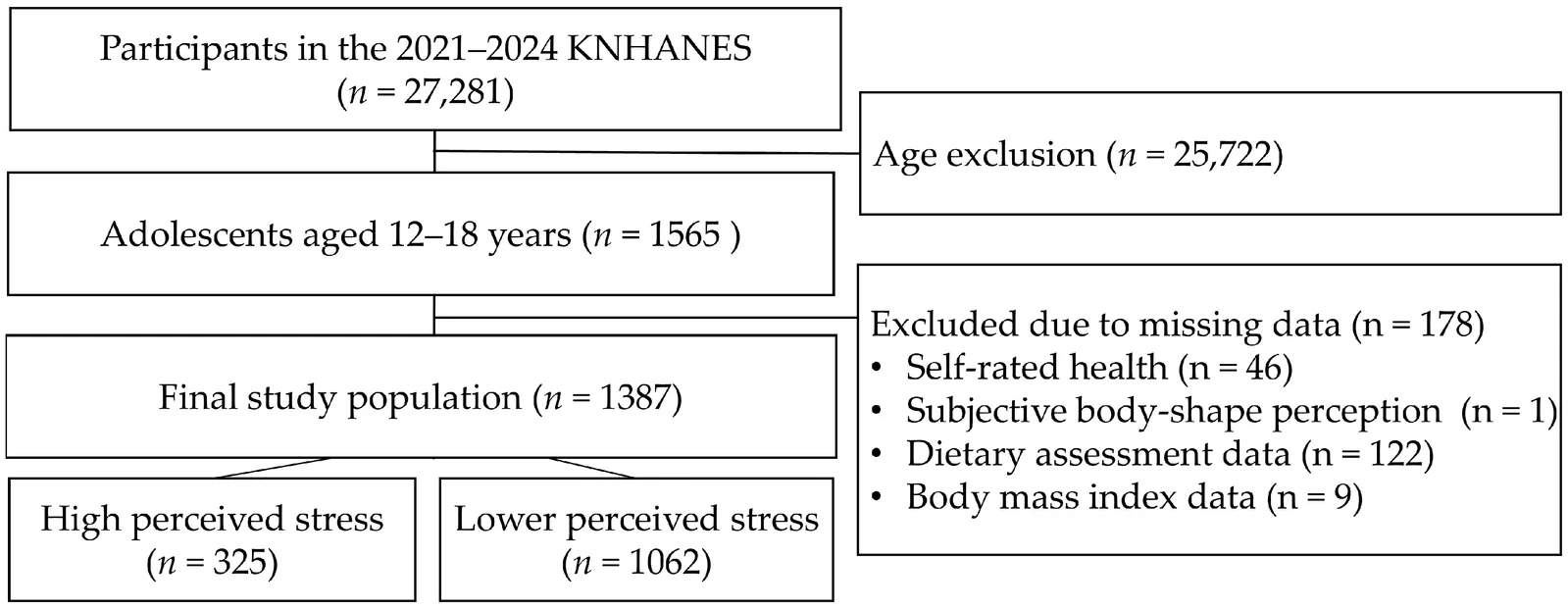
Inclusion and exclusion of participants.

This cross-sectional study used data from the 2021–2024 Korea National Health and Nutrition Examination Survey (KNHANES) to examine combined patterns of stress and subjective body shape and their relationships with health-related characteristics among adolescents. KNHANES is a nationwide survey conducted to assess health status, health behaviors, and food and nutrient intake. Of 27,281 participants in the 2021–2024 KNHANES, 1565 adolescents aged 12–18 years were initially identified. The age range of 12–18 years was selected in accordance with previous studies using KNHANES data that included Korean adolescents aged 12–18 years [14,15]. Of these, 178 adolescents with missing data for variables required for the analysis were excluded, resulting in a final analytic sample of 1387 adolescents. The excluded participants had missing data on self-rated health (n = 46), subjective body-shape perception (n = 1), dietary assessment data (n = 122), or body mass index (n = 9). A common complete-case sample was used across analyses to maintain a consistent analytic population, including analyses of meal frequency and nutrient intake and the exploratory Random Forest/SHAP analysis. Therefore, participants with missing dietary assessment data were excluded, although these variables were not included in the adjustment sets of the primary regression models. No imputation was performed. Detailed participant selection is presented in Figure 1. Written informed consent was obtained in accordance with the KNHANES consent procedures. For participants younger than 18 years, informed consent was obtained from their parents or legal guardians. The present study was a secondary analysis of de-identified KNHANES data and was approved by the Institutional Review Board of Gangneung-Wonju National University (GWNUIRB-R2020-16; approval date: 18 March 2020).

### 2.2. Stress, Subjective Body Shape, and Health-Related Questionnaire Variables

Perceived stress was assessed using the KNHANES item on the usual level of perceived stress. This dichotomization was used because the analysis focused on identifying characteristics of adolescents with high perceived stress; therefore, the moderate, low, and very low categories were combined as the reference group [16]. Response options were: (1) very much, (2) much, (3) a little, and (4) almost none. Participants who responded “very much” or “much” were classified as high stress (HS), whereas those who responded “a little” or “almost none” were classified as low stress (LS) [17]. Responses coded as not applicable or unknown/nonresponse were treated as missing. Subjective body shape was assessed using the KNHANES item on self-perceived body shape. Response options were: (1) very thin, (2) slightly thin, (3) normal, (4) slightly overweight, and (5) very overweight. Responses of 1–3 were classified as self-perceived non-overweight, whereas responses of 4–5 were classified as self-perceived overweight. Responses coded as not applicable or unknown/nonresponse were treated as missing.

Self-assessed health-related questionnaire variables included depressive symptoms and self-rated health. Depressive symptoms were assessed using a single KNHANES screening item asking whether participants had experienced sadness or hopelessness continuously for at least two weeks. Responses were classified as yes or no, and not applicable or unknown/nonresponse responses were treated as missing. This measure was treated as a self-reported depressive symptom indicator and not as a clinical diagnosis of depression. Self-rated health was assessed using five response options: (1) very good, (2) good, (3) fair, (4) poor, and (5) very poor. Responses of 1–3 were classified as favorable self-rated health, whereas responses of 4–5 were classified as poor self-rated health. Unknown/nonresponse was treated as missing [18]. Lifetime alcohol experience was assessed by asking whether participants had ever consumed alcohol. Responses were classified as no or yes, and not applicable or unknown/nonresponse responses were treated as missing. Lifetime smoking experience was assessed based on cigarette smoking history. Participants who reported smoking fewer than 100 cigarettes or 100 cigarettes or more during their lifetime were classified as having smoking experience, whereas those who had never smoked were classified as having no smoking experience. Unknown/nonresponse responses were treated as missing.

Physical activity was assessed as the number of days during the past 7 days on which participants engaged in at least 60 min of physical activity. It was classified as high (≥3 days/week) or low (<3 days/week). Exercise intensity was not separately assessed. Strengthening exercise was assessed as the number of days of strength exercise during the past week and was classified as high (≥2 days/week) or low (<2 days/week) [19]. Household income was assessed as total monthly household income and was recorded as a continuous variable in units of 10,000 Korean won (KRW); it was not equivalized for household size.

### 2.3. Subjective Body Shape and Combined Stress–Body-Shape Joint Categories

Subjective body shape was classified into self-perceived non-overweight and self-perceived overweight categories and combined with stress (high or low) to construct four joint categories: G1, low stress + self-perceived non-overweight (reference group); G2, high stress + self-perceived non-overweight; G3, low stress + self-perceived overweight; and G4, high stress + self-perceived overweight. These joint categories were used to compare prespecified health-related outcomes across the observed combinations of perceived stress and self-perceived body shape and were not intended to represent statistical interaction or synergistic effects.

### 2.4. Objective Weight Status and Subjective Body Shape

Body mass index (BMI) was calculated from measured height and weight. Objective weight status was determined using the HE_BMI_pct variable provided in the KNHANES raw dataset, which was classified according to age and sex with reference to the 2017 Korean National Growth Charts for Children and Adolescents [15]. Weight status was classified using sex- and age-specific BMI percentiles as underweight (<5th percentile), normal weight (≥5th to <85th percentile), overweight (≥85th to <95th percentile), and obesity (≥95th percentile) [20]. For the present analysis, underweight and normal weight were combined as non-overweight, whereas overweight and obesity were combined as overweight/obesity. These objective weight-status categories were combined with subjective body-shape perception to define four groups: G1, objective non-overweight + self-perceived non-overweight (concordant non-overweight); G2, objective non-overweight + self-perceived overweight (overestimation); G3, objective overweight/obesity + self-perceived overweight (concordant overweight); and G4, objective overweight/obesity + self-perceived non-overweight (underestimation).

### 2.5. Nutrient Intake

Nutritional characteristics included total energy intake (kcal/day), protein (g/day), fat (g/day), and carbohydrate (g/day). Daily absolute intake of each nutrient was used when comparing participant characteristics according to stress level. For the SHAP analysis, protein, fat, and carbohydrate intakes were adjusted for total energy intake using the residual method. Each nutrient was regressed on total energy intake, and the residual was added to the mean nutrient intake to obtain the energy-adjusted value [21]. Breakfast, lunch, and dinner frequency were assessed based on the usual weekly frequency of each meal during the past year. Response options were 5–7, 3–4, 1–2, or 0 times/week. Each meal was classified as high frequency (5–7 times/week) or low frequency (0–4 times/week), and unknown/nonresponse was treated as missing. Low frequency indicated less frequent consumption of the corresponding meal; low lunch frequency was used as the corresponding variable in the SHAP analysis.

### 2.6. Statistical Analysis

Because KNHANES uses a complex, multistage, stratified sampling design, survey weights, strata, and primary sampling units (PSUs) were incorporated into descriptive analyses, hypothesis tests, and logistic regression models. For the pooled 2021–2024 data, the year-specific survey weights were divided by four to obtain combined weights, following the KNHANES guidelines. Continuous variables are presented as survey-weighted means ± standard errors (SEs), and categorical variables as unweighted frequencies with survey-weighted percentages [n (%)]. For comparisons between the high- and low-stress groups in Table 1, survey-weighted linear regression was used for continuous variables, whereas survey-weighted chi-square tests were used for categorical variables.

The four groups combining stress and subjective body shape were examined for depressive symptoms, poor self-rated health, and alcohol experience using survey-weighted multivariable logistic regression. Depressive symptoms, poor self-rated health, and alcohol experience were prespecified as health-related outcomes based on the study objectives and prior evidence and were not selected according to statistical significance in Table 1. The comparisons in Table 1 were considered descriptive and were not used to select variables for subsequent regression analyses. No universal adjustment for multiple comparisons was applied because the regression analyses addressed prespecified outcomes, whereas the Random Forest/SHAP analysis was considered exploratory. Effect estimates and 95% confidence intervals were emphasized when interpreting the regression results. The joint stress–body-shape category analysis was considered the primary analysis, the objective–subjective weight-status analysis was considered secondary, and the Random Forest/SHAP analysis was considered exploratory. Adjusted odds ratios (AORs) and 95% confidence intervals (CIs) were calculated after adjustment for age, sex, household income, and BMI; sex-stratified models excluded sex. These covariates were selected based on their substantive relevance as demographic and weight-related factors rather than statistical significance in the descriptive analyses. Formal sex-by-joint category interaction terms were tested for each outcome.

For the objective–subjective weight-status analysis, the four concordance/discordance groups were treated as the exposure, high perceived stress as the outcome, and G1 (concordant non-overweight) as the reference group. Models were adjusted for age, sex, and household income; BMI was not additionally adjusted for because objective weight status was itself a component of the exposure classification. Sample sizes represent unweighted counts, whereas population estimates and inferential statistics account for the complex survey design. Model diagnostics were assessed for multicollinearity, separation, convergence, and the functional form of continuous covariates. Because a nonlinear relationship between BMI and poor self-rated health was identified, a sensitivity analysis including a quadratic BMI term was additionally performed.

Random Forest analysis was performed as an exploratory cross-sectional classification analysis of high perceived stress. Candidate predictors included demographic, body-shape, health, lifestyle, dietary, and energy-adjusted nutrient variables. For the Random Forest analysis, complete cases were used without imputation, feature scaling, or class-balancing resampling. Hyperparameter tuning was performed using 3-fold cross-validation within each training fold of an outer 5-fold stratified cross-validation. Model performance was evaluated exclusively on the corresponding held-out outer fold, thereby ensuring that observations used for performance evaluation were not used during hyperparameter tuning. The final hyperparameters were 300 trees, a maximum tree depth of 3, square-root feature selection at each split, and a minimum of 10 samples per leaf. SHAP values were calculated for held-out observations to reduce optimistic interpretation of feature contributions. The 95% CIs for the weighted mean absolute SHAP values were estimated using 1000 participant-level bootstrap resamples. Survey sampling weights were incorporated when calculating the weighted mean absolute SHAP value within each bootstrap sample, whereas survey strata and primary sampling units were not incorporated into the bootstrap resampling procedure. Because survey strata and PSUs were not incorporated into the Random Forest/SHAP procedure, these analyses were considered exploratory. Analyses were performed using IBM SPSS Statistics version 26.0 (IBM Corp., Armonk, NY, USA) and Python version 3.12 (Python Software Foundation, Wilmington, DE, USA), with two-sided *p* < 0.05 considered statistically significant.

## 3. Results

### 3.1. Participant Characteristics According to Perceived Stress Level

Among the 1387 adolescents, 325 were classified into the HS group and 1062 into the LS group (Table 1). Among the 1565 age-eligible adolescents, 1387 were included in the final analysis and 178 were excluded because of missing data. The mean age was 14.68 years among included adolescents (n = 1387) and 14.93 years among excluded adolescents (n = 178). The proportion of males was 51.6% (715/1387) in the included group and 52.8% (94/178) in the excluded group. Overall, the included and excluded adolescents showed similar distributions of age and sex, with no substantial differences in these observed demographic characteristics. The HS group showed higher weighted proportions of self-perceived overweight (42.0% vs. 31.0%), depressive symptoms (25.4% vs. 2.1%), poor self-rated health (14.1% vs. 5.3%), alcohol experience (25.8% vs. 17.5%), and low lunch frequency (13.7% vs. 6.4%) than the LS group (all *p* < 0.05). No significant between-group differences were observed in age, sex, household income, BMI, smoking experience, aerobic physical activity, strengthening exercise, breakfast or dinner frequency, or nutrient intake (all *p* > 0.05).

### 3.2. Combined Perceived Stress and Subjective Body-Shape Joint Categories and Health-Related Outcomes

Four joint categories were defined based on perceived stress and subjective body-shape perception. Depressive symptoms, poor self-rated health, and alcohol experience were examined as prespecified health-related outcomes (Table 2). In the overall analysis, G4 showed significantly higher odds of depressive symptoms (AOR, 15.96; 95% CI, 6.52–39.10), poor self-rated health (AOR, 7.55; 95% CI, 3.22–17.73), and alcohol experience (AOR, 3.04; 95% CI, 1.31–7.07) compared with G1. G2 showed significantly higher odds of depressive symptoms (AOR, 14.67; 95% CI, 7.48–28.79) and poor self-rated health (AOR, 2.78; 95% CI, 1.23–6.29), whereas G3 showed significantly higher odds only for poor self-rated health (AOR, 2.68; 95% CI, 1.28–5.61). In the sex-stratified analyses, G4 showed significantly higher odds of depressive symptoms and poor self-rated health in both boys and girls. For alcohol experience, significantly higher odds in G4 were observed in girls (AOR, 6.99; 95% CI, 2.36–20.71), but not in boys. However, formal sex-by-joint category interaction tests were not significant for depressive symptoms (*p* = 0.695), poor self-rated health (*p* = 0.444), or alcohol experience (*p* = 0.268), indicating no statistical evidence that the joint category estimates differed by sex. A nonlinear relationship between BMI and poor self-rated health was identified (*p* < 0.001). In a sensitivity analysis including a quadratic BMI term, the direction and statistical significance of the joint category estimates were unchanged, although the magnitude of some estimates differed (G2: AOR, 2.70; 95% CI, 1.21–6.01; G3: AOR, 4.85; 95% CI, 2.11–11.14; G4: AOR, 15.83; 95% CI, 6.32–39.66).

### 3.3. Objective–Subjective Weight-Status Groups and High Perceived Stress

**Figure 2:**
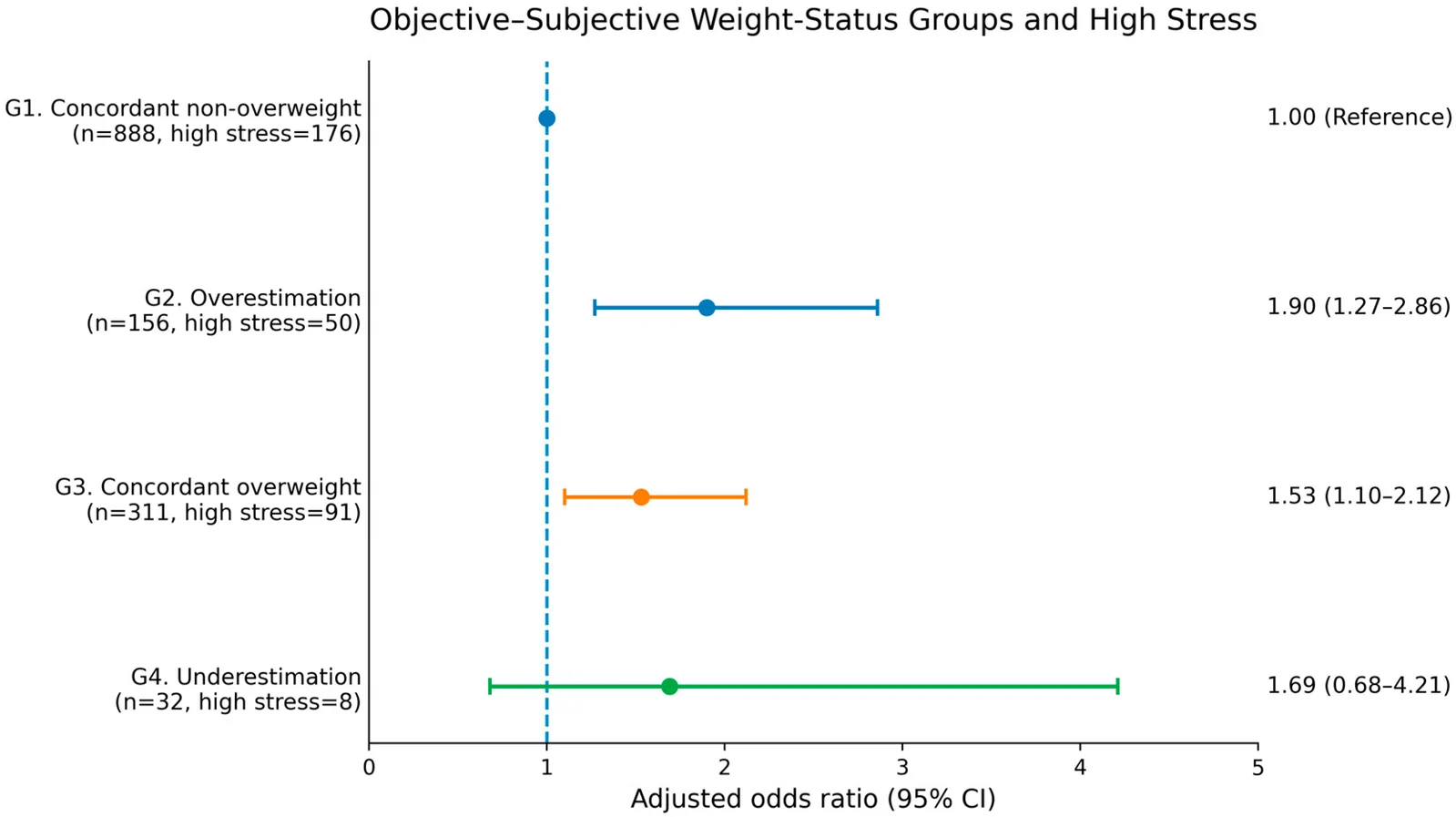
Adjusted Odds Ratios for High Perceived Stress Across Objective–Subjective Weight-Status Groups. Groups: G1, objective non-overweight + self-perceived non-overweight (concordant non-overweight); G2, objective non-overweight + self-perceived overweight (overestimation); G3, objective overweight/obesity + self-perceived overweight (concordant overweight); G4, objective overweight/obesity + self-perceived non-overweight (underestimation). Dots represent adjusted odds ratios (AORs), and horizontal lines indicate 95% confidence intervals (CIs). The vertical dashed line indicates the reference value (OR = 1.0). Different colors distinguish the weight-status groups and do not represent additional statistical information.

The AORs for high perceived stress across the four objective–subjective weight-status groups are presented in Figure 2. Using G1 (objective non-overweight + self-perceived non-overweight) as the reference group, G2 (objective non-overweight + self-perceived overweight) showed significantly higher odds of high perceived stress (AOR, 1.90; 95% CI, 1.27–2.86; *p* = 0.002). G3 (objective overweight/obesity + self-perceived overweight) also showed significantly higher odds (AOR, 1.53; 95% CI, 1.10–2.12; *p* = 0.011). In contrast, G4 (objective overweight/obesity + self-perceived non-overweight) did not show significantly higher odds (AOR, 1.69; 95% CI, 0.68–4.21; *p* = 0.261). These estimates represent comparisons of the observed objective–subjective weight-status combinations with G1 and should not be interpreted as independent effects of weight-status discordance.

### 3.4. Exploratory Random Forest and SHAP Analysis of High Perceived Stress

The Random Forest model was evaluated as an exploratory cross-sectional classification model for high perceived stress. Using nested cross-validation, the model showed modest classification performance, with a ROC-AUC of 0.615, a precision–recall AUC (PR-AUC) of 0.438, and a Brier score of 0.218. Accordingly, the SHAP analysis was interpreted as an exploratory assessment of feature contributions to high-stress classification rather than as evidence of prospective predictive performance or causal effects.

**Figure 3:**
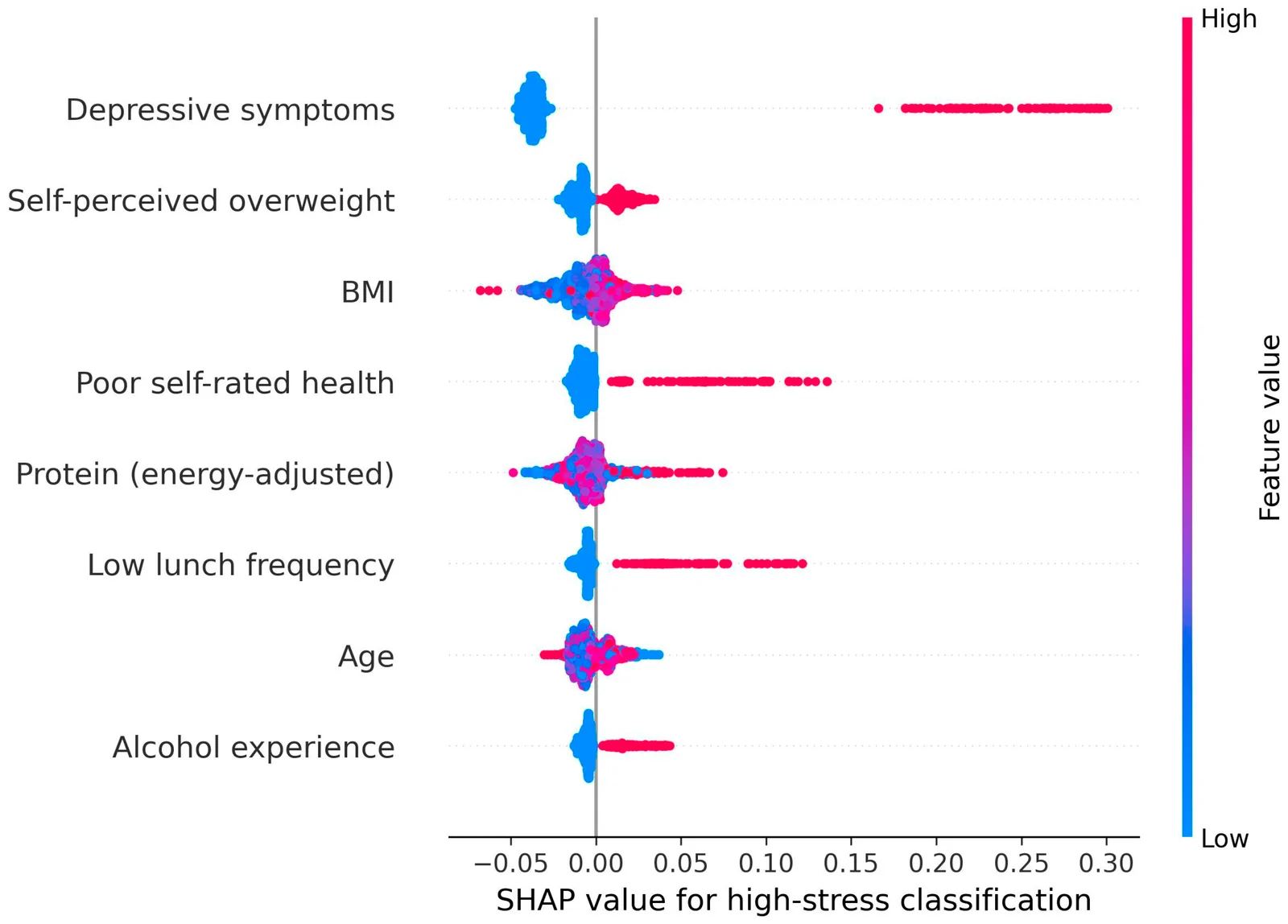
SHAP Summary Plot for High Stress. Red and blue indicate higher and lower feature values, respectively. Positive SHAP values indicate contributions toward high-stress classification, whereas negative values indicate contributions away from high-stress classification. SHAP values are shown for the high-stress class on the probability scale. Sampling weights were incorporated during model fitting.

SHAP-based feature importance is presented in Table 3 and Figure 3. Depressive symptoms showed the largest mean absolute SHAP value, followed by self-perceived overweight, poor self-rated health, BMI, energy-adjusted protein intake, low lunch frequency, age, and alcohol experience. Relative contributions were calculated from the summed mean absolute SHAP values among the eight displayed variables.

The SHAP summary plot illustrates both the magnitude and direction of individual feature contributions to the fitted classification model (Figure 3). These SHAP values represent contributions to model classification and should not be interpreted as independent effect estimates or causal relationships. Given the modest out-of-sample classification performance, the Random Forest/SHAP findings should be considered complementary and exploratory.

## 4. Discussion

In this study, health-related outcomes differed across the joint categories of perceived stress and subjective body-shape perception among Korean adolescents. Adolescents with high stress and self-perceived overweight showed higher odds of depressive symptoms, poor self-rated health, and alcohol experience than those with low stress and self-perceived non-overweight. In the secondary analysis, adolescents with objective non-overweight status who perceived themselves as overweight showed higher odds of high perceived stress than those with concordant non-overweight status.

Subjective body shape in adolescents partly reflects self-evaluation of the body and psychological status. The pattern observed for depressive symptoms in this study is relatively consistent with previous research. Even among adolescents with normal weight, perceiving oneself as overweight may be accompanied by more frequent depressive symptoms. In a meta-analysis, Haynes et al. reported that perceived overweight was associated with 1.42-fold higher depressive symptoms and a 1.41-fold higher tendency toward suicidality, even after objective weight status was considered [22]. Adolescent studies have also shown that how individuals perceive their weight may correspond more closely with mental health indicators, including perceived stress and depressed mood, than actual weight status. In particular, girls who perceived themselves as overweight regardless of actual weight status were more likely to experience depressed mood and stress, whereas boys who perceived themselves as underweight were more likely to experience suicidal ideation [23,24]. In a study of 109,373 middle-school students, depressive symptoms were more frequent among girls with normal weight who perceived themselves as overweight [4]. In the present study, higher odds of depressive symptoms were observed in both high-stress categories (G2 and G4), whereas G3 did not show higher odds than G1. These findings should be interpreted as differences among the observed joint categories rather than as evidence of an independent or additional effect of subjective body-shape perception.

Self-rated health can be viewed as a self-assessment encompassing emotional and social experiences and overall well-being, and the differences observed in poor self-rated health may be interpreted in this context [25]. A longitudinal study reported that body and weight concerns among girls were associated with self-esteem at follow-up [26]. In a Korean adolescent study by Choi et al., the relationship between self-perceived weight and poor self-rated health differed according to actual BMI, and poor self-rated health was more frequent among adolescents with overestimation [27]. In the present study, joint categories of stress and subjective body shape were used to descriptively compare multiple health-related outcomes. This difference may partly reflect the broader nature of self-rated health, which encompasses physical, psychological, behavioral, and social dimensions of perceived health [28], whereas depressive symptoms represent a more specific psychological outcome. Thus, the findings indicate that the pattern observed for G3 differed according to the health-related outcome examined and should not be interpreted as a uniform relationship across outcomes.

Alcohol use during adolescence is a health-risk behavior influenced by multiple factors, including peer relationships, social environment, and stress coping; therefore, a direct causal relationship with subjective body shape should not be assumed. A longitudinal study reported that higher stress may be linked to alcohol-related problems in adolescents and that coping strategies and sex may influence the relationship between stress and alcohol use [29]. Thus, the present findings should not be interpreted as indicating that body-shape perception directly causes alcohol use, but rather that health-risk behaviors may coexist in some adolescents with both high stress and negative body self-evaluation. Previous studies reported that weight overestimation was accompanied by greater alcohol experience among girls but not boys [30], and Lee et al. found that boys with concordant weight perception were less likely to drink and smoke, whereas girls with overestimation had 1.107-fold higher alcohol use and 1.130-fold higher smoking [11]. Although sex-stratified patterns differed descriptively, formal interaction tests did not show statistically significant evidence of differences in these associations by sex.

In the concordance and discordance analysis of objective weight status and subjective body shape, underestimation was not significantly related to HS, whereas overestimation was significant. However, the non-significant finding for underestimation should be interpreted cautiously because the subgroup was relatively small and the estimate had a wide confidence interval, indicating limited statistical precision. Thus, the differing findings for overestimation and underestimation may partly reflect differences in subgroup size and precision rather than distinct psychological patterns alone. This pattern is similar to previous findings. In a study of Chinese adolescents, overweight misperception was more common among girls, and perceived overweight was more closely linked to adverse psychological states, including anxiety and depression [31]. Riahi et al. also reported a weight-misperception prevalence of 59.1%, with increased risks of worthlessness, worry, aggression, insomnia, or depression among adolescents with overestimation or underestimation [12]. However, direct comparison with these studies should be made cautiously because of differences in the measurement and classification of body-shape perception, participant age and sex composition, study design, and cultural context [12,31].

Regarding dietary behavior, meal frequency was also associated with stress in the present study. Lee et al., using KNHANES data from 1413 Korean adolescents, examined meal skipping and mental health and reported that breakfast skipping was associated with increased stress and depressed mood, while a greater number of skipped meals per day was accompanied by more frequent stress, depressed mood, and suicidal ideation [32]. The differences in lunch and dinner frequency observed in the present study may therefore reflect irregular eating patterns occurring alongside adolescents’ psychological status rather than an effect attributable to any specific meal.

However, the direction between meal frequency and stress cannot be determined. Similar patterns have been observed in adolescents from other countries. Breakfast skipping explained significant differences in depressive symptoms [33], adolescents who regularly had family dinners showed fewer mental health problems, including anxiety and insomnia [34], and more frequent breakfast skipping was accompanied by greater psychiatric distress [35]. These findings suggest that the observed differences in lunch and dinner frequency may reflect irregular eating behaviors occurring alongside adolescents’ psychological status rather than effects of specific meals. Given the cross-sectional design, the observed differences should not be interpreted causally. Moreover, given the absence of clear differences in major nutrient intakes between the HS and LS groups, behavioral aspects such as meal regularity or timing may warrant further investigation when examining stress and dietary patterns.

SHAP analysis complemented the conventional analyses by providing information on the relative contribution of individual features to the classification of HS and LS. Unlike conventional statistical comparisons, SHAP values describe how individual features contribute to model predictions and should not be interpreted as measures of statistical significance or causality [36]. Self-perceived overweight, poor self-rated health, alcohol experience, and low lunch frequency were among the features contributing most strongly to the model’s classification of HS and LS, broadly corresponding to patterns observed in the conventional analyses. In contrast, energy-adjusted nutrients showed no significant differences between the HS and LS groups and did not show a consistent directional pattern in the SHAP results. Thus, the SHAP findings provide complementary information about the relative predictive contribution of these characteristics but do not establish independent or causal relationships with stress [37].

Unlike previous studies, this study constructed joint categories combining stress and subjective body shape and evaluated multiple health-related outcomes together. Another strength is the separate assessment of concordance and discordance between objective weight status and subjective body shape and the complementary use of Random Forest-based SHAP analysis to explore the relative contributions of health, lifestyle, and nutritional characteristics to HS classification.

Nevertheless, this study has several limitations. First, because of the cross-sectional design, temporal or causal relationships among stress, subjective body shape, and health-related characteristics cannot be determined. Second, the complete-case analysis may have introduced selection bias due to the exclusion of participants with missing data. In addition, the use of a common complete-case sample may have excluded some adolescents who otherwise had sufficient data for the primary regression analyses, resulting in a loss of otherwise eligible observations. Some estimates, particularly in the sex-stratified analyses, had wide confidence intervals because of small subgroup and outcome-event counts and should therefore be interpreted with caution. Third, several variables were dichotomized, which may have resulted in loss of information, and key variables were assessed using standardized KNHANES self-reported items, including single-item measures, and may therefore be subject to recall, reporting, and classification errors. In particular, depressive symptoms were assessed using a single screening item rather than a validated diagnostic instrument and should not be interpreted as a clinical diagnosis of depression. Fourth, residual and unmeasured confounding remains possible because factors such as academic burden, peer and family environments, body dissatisfaction, weight-control behaviors, sleep, and social media use were not fully considered. Fifth, potential differences across survey years and uncertainty in weight-status classification may have affected the estimates. Sixth, the Random Forest and SHAP analyses were exploratory and internally evaluated without external validation; therefore, their findings should not be interpreted as independent effects, causal influences, or externally validated predictive performance. In addition, although survey sampling weights were incorporated, survey strata and PSUs were not incorporated into the Random Forest/SHAP procedure, and the bootstrap CIs should therefore be interpreted as exploratory uncertainty estimates rather than design-based population confidence intervals. Finally, because the study included Korean adolescents, the findings may not be generalizable to adolescents in other countries or cultural contexts. Future longitudinal studies with external validation are warranted to further investigate the observed patterns.

## 5. Conclusions

In this study, health-related outcomes differed across joint categories of perceived stress and subjective body-shape perception among Korean adolescents. Adolescents with high stress and self-perceived overweight showed higher odds of depressive symptoms, poor self-rated health, and alcohol experience than those with low stress and self-perceived non-overweight. In the secondary analysis, adolescents with objective non-overweight status who perceived themselves as overweight showed higher odds of high stress than those with concordant non-overweight status.

## Figures and Tables

**Table 1:** Characteristics of Participants According to Perceived Stress Level.

Variables	High Stress (n = 325)	Low Stress (n = 1062)	*p*
Age, years	15.1 ± 0.1	14.9 ± 0.1	0.274
Sex, n (%), Boys/Girls	155 (47.1)/170 (52.9)	560 (53.5)/502 (46.5)	0.058
Household income, 10,000 KRW	661.4 ± 23.3	664.2 ± 14.8	0.905
Body mass index, kg/m^2^	22.0 ± 0.3	21.7 ± 0.2	0.270
Subjective self-assessment, n (%)			
Self-perceived body shape, non-overweight/overweight	184 (58.0)/143 (42.0)	736 (69.0)/326 (31.0)	<0.001
Depressive symptoms, No/Yes	250 (74.6)/77 (25.4)	1039 (97.9)/23 (2.1)	<0.001
Self-rated health, favorable/poor	282 (85.9)/45 (14.1)	1011 (94.7)/51 (5.3)	<0.001
Health lifestyle behaviors, n (%)			
Lifetime alcohol experience, No/Yes	254 (74.2)/73 (25.8)	913 (82.5)/149 (17.5)	0.005
Lifetime smoking experience, No/Yes	304 (91.5)/23 (8.5)	1006 (93.8)/56 (6.2)	0.212
Aerobic physical activity, high/low	116 (33.8)/211 (66.2)	342 (32.1)/720 (67.9)	0.657
Strengthening exercise, high/low	62 (20.6)/263 (79.4)	197 (19.1)/865 (80.9)	0.575
Breakfast frequency, high/low	151 (46.3)/174 (53.7)	549 (51.0)/513 (49.0)	0.207
Lunch frequency, high/low	284 (86.3)/41 (13.7)	997 (93.6)/65 (6.4)	<0.001
Dinner frequency, high/low	286 (88.5)/39 (11.5)	977 (90.9)/85 (9.1)	0.286
Dietary intake			
Total energy intake, kcal/day	1898.6 ± 45.6	1936.3 ± 27.3	0.464
Protein, g/day	74.1 ± 2.1	75.1 ± 1.3	0.669
Fat, g/day	58.2 ± 2.1	59.0 ± 1.1	0.720
Carbohydrate, g/day	264.9 ± 6.1	271.1 ± 4.0	0.388

Values are presented as unweighted n with survey-weighted percentages or survey-weighted means ± SE. Statistical tests accounted for the complex survey design. KRW, Korean won.

**Table 2:** Odds ratio of Combined Stress and Subjective Body-Shape Groups with Depressive Symptoms, Self-Rated Health, and Alcohol Experience.

Outcome	G1: LS + Non-Overweight Reference	G2: HS + Non-Overweight AOR (95% CI)	G3: LS + Overweight AOR (95% CI)	G4: HS + Overweight AOR (95% CI)
Overall, n = 1387	n = 736	n = 184	n = 326	n = 141
Depressive symptoms	15; 1.00	42; 14.67 (7.48–28.79)	8; 0.91 (0.28–2.97)	35; 15.96 (6.52–39.10)
Poor self-rated health	21; 1.00	15; 2.78 (1.23–6.29)	30; 2.68 (1.28–5.61)	30; 7.55 (3.22–17.73)
Alcohol experience	103; 1.00	41; 1.47 (0.87–2.47)	46; 1.15 (0.60–2.20)	32; 3.04 (1.31–7.07)
Boys, n = 715	n = 377	n = 89	n = 183	n = 66
Depressive symptoms	8; 1.00	19; 13.37 (4.84–36.89)	3; 0.47 (0.08–2.87)	17; 16.17 (4.24–61.60)
Poor self-rated health	14; 1.00	7; 2.50 (0.86–7.30)	19; 2.19 (0.87–5.54)	12; 4.19 (1.40–12.58)
Alcohol experience	71; 1.00	22; 1.22 (0.60–2.49)	31; 0.87 (0.37–2.04)	13; 1.27 (0.41–3.96)
Girls, n = 672	n = 359	n = 95	n = 143	n = 75
Depressive symptoms	7; 1.00	23; 16.52 (6.71–40.71)	5; 1.54 (0.33–7.24)	18; 16.71 (4.84–57.65)
Poor self-rated health	7; 1.00	8; 3.16 (0.95–10.50)	11; 3.29 (1.06–10.18)	18; 15.14 (4.68–48.97)
Alcohol experience	32; 1.00	19; 1.87 (0.86–4.09)	15; 1.69 (0.70–4.09)	19; 6.99 (2.36–20.71)

CI, confidence interval; AOR, adjusted odds ratio. Values in outcome rows are presented as the number of events followed by AOR (95% CI). Adjustment: The overall analysis was adjusted for age, sex, household income, and BMI; the sex-stratified analyses for boys and girls were adjusted for age, household income, and BMI. Groups: G1: low stress + self-perceived non-overweight (reference group)/G2: high stress + self-perceived non-overweight/G3: low stress + self-perceived overweight/G4: high stress + self-perceived overweight.

**Table 3:** SHAP-Based Feature Importance for High-Stress Classification.

Predictor	Weighted Mean |SHAP|	95% CI	Relative Contribution Among Displayed Variables (%)
Depressive symptoms	0.0529	0.0499–0.0559	33.6
Self-perceived overweight	0.0116	0.0113–0.0119	7.3
Poor self-rated health	0.0113	0.0103–0.0124	7.2
BMI	0.0111	0.0106–0.0116	7.1
Protein (energy-adjusted)	0.0106	0.0099–0.0111	6.7
Low lunch frequency	0.0100	0.0092–0.0110	6.4
Age	0.0084	0.0082–0.0088	5.4
Alcohol experience	0.0084	0.0080–0.0089	5.4

Abbreviations: SHAP, SHapley Additive exPlanations; CI, confidence interval. Weighted mean |SHAP| represents the survey-weighted mean absolute SHAP value. The 95% CIs were estimated using 1000 participant-level bootstrap resamples, with survey sampling weights incorporated when calculating the weighted mean absolute SHAP value within each bootstrap sample. Survey strata and primary sampling units were not incorporated into the bootstrap resampling procedure. Relative contribution was calculated as the percentage of the total weighted mean |SHAP| among the eight displayed features. Accordingly, these 95% CIs should be interpreted as exploratory uncertainty estimates rather than design-based population confidence intervals.

## Data Availability

Publicly available datasets were analyzed in this study. The data are available from the Korea National Health and Nutrition Examination Survey database at https://knhanes.kdca.go.kr/knhanes/eng/index.do (accessed on 1 April 2026).
